# Using Domain Adaptation and Inductive Transfer Learning to Improve Patient Outcome Prediction in the Intensive Care Unit: Retrospective Observational Study

**DOI:** 10.2196/52730

**Published:** 2024-08-21

**Authors:** Maruthi Kumar Mutnuri, Henry Thomas Stelfox, Nils Daniel Forkert, Joon Lee

**Affiliations:** 1 Data Intelligence for Health Lab Cumming School of Medicine University of Calgary Calgary, AB Canada; 2 Department of Biomedical Engineering Schulich School of Engineering University of Calgary Calgary, AB Canada; 3 Department of Critical Care Medicine Cumming School of Medicine University of Calgary Calgary, AB Canada; 4 O’Brien Institute for Public Health Cumming School of Medicine University of Calgary Calgary, AB Canada; 5 Department of Radiology Cumming School of Medicine University of Calgary Calgary, AB Canada; 6 Alberta Children’s Hospital Research Institute Cumming School of Medicine University of Calgary Calgary, AB Canada; 7 Department of Cardiac Sciences Cumming School of Medicine University of Calgary Calgary, AB Canada; 8 Department of Community Health Sciences Cumming School of Medicine University of Calgary Calgary, AB Canada; 9 Department of Preventive Medicine School of Medicine Kyung Hee University Seoul Republic of Korea

**Keywords:** transfer learning, patient outcome prediction, intensive care, deep learning, electronic health record

## Abstract

**Background:**

Accurate patient outcome prediction in the intensive care unit (ICU) can potentially lead to more effective and efficient patient care. Deep learning models are capable of learning from data to accurately predict patient outcomes, but they typically require large amounts of data and computational resources. Transfer learning (TL) can help in scenarios where data and computational resources are scarce by leveraging pretrained models. While TL has been widely used in medical imaging and natural language processing, it has been rare in electronic health record (EHR) analysis. Furthermore, domain adaptation (DA) has been the most common TL method in general, whereas inductive transfer learning (ITL) has been rare. To the best of our knowledge, DA and ITL have never been studied in-depth in the context of EHR-based ICU patient outcome prediction.

**Objective:**

This study investigated DA, as well as rarely researched ITL, in EHR-based ICU patient outcome prediction under simulated, varying levels of data scarcity.

**Methods:**

Two patient cohorts were used in this study: (1) eCritical, a multicenter ICU data from 55,689 unique admission records from 48,672 unique patients admitted to 15 medical-surgical ICUs in Alberta, Canada, between March 2013 and December 2019, and (2) Medical Information Mart for Intensive Care III, a single-center, publicly available ICU data set from Boston, Massachusetts, acquired between 2001 and 2012 containing 61,532 admission records from 46,476 patients. We compared DA and ITL models with baseline models (without TL) of fully connected neural networks, logistic regression, and lasso regression in the prediction of 30-day mortality, acute kidney injury, ICU length of stay, and hospital length of stay. Random subsets of training data, ranging from 1% to 75%, as well as the full data set, were used to compare the performances of DA and ITL with the baseline models at various levels of data scarcity.

**Results:**

Overall, the ITL models outperformed the baseline models in 55 of 56 comparisons (all *P* values <.001). The DA models outperformed the baseline models in 45 of 56 comparisons (all *P* values <.001). ITL resulted in better performance than DA in terms of the number of times and the margin with which it outperformed the baseline models. In 11 of 16 cases (8 of 8 for ITL and 3 of 8 for DA), TL models outperformed baseline models when trained using 1% data subset.

**Conclusions:**

TL-based ICU patient outcome prediction models are useful in data-scarce scenarios. The results of this study can be used to estimate ICU outcome prediction performance at different levels of data scarcity, with and without TL. The publicly available pretrained models from this study can serve as building blocks in further research for the development and validation of models in other ICU cohorts and outcomes.

## Introduction

Electronic health records (EHRs) are databases that hospitals and health care providers use to record an individual’s health history. There has been significant progress in using deep learning models for predicting patient outcomes using EHR data [1]. However, using deep learning models is not feasible in some settings, such as rural hospital ICUs, which have low patient volumes and limited computational capacity due to budget restrictions.

**Figure 1:**
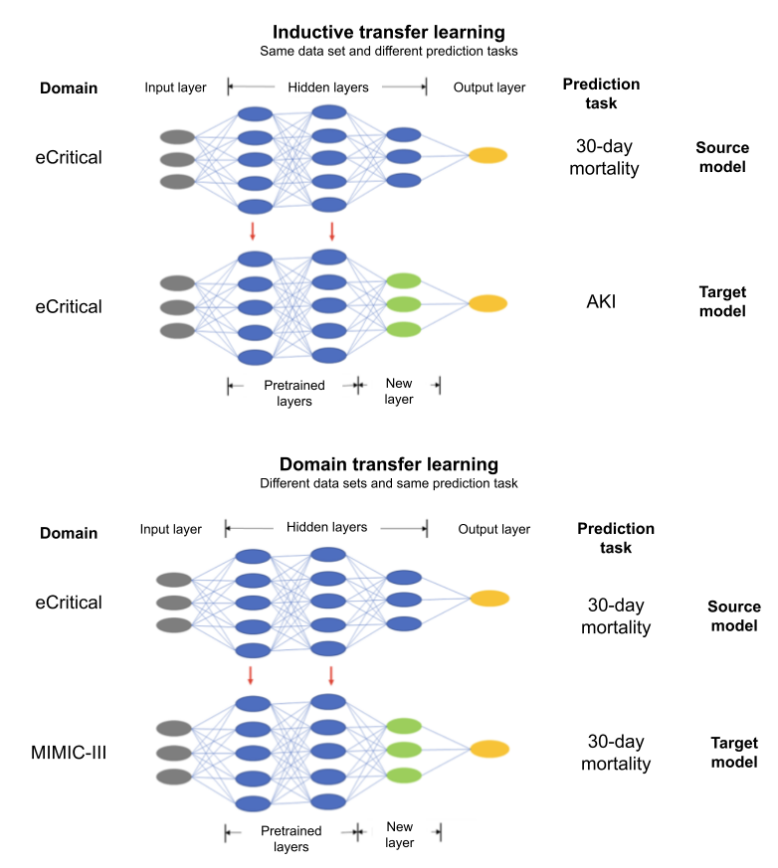
An illustration of how DA and ITL were applied in this study. For ITL, the source and target prediction tasks were 30-day mortality and AKI, respectively, while the source and target domains were both eCritical. For DA, the source and target prediction tasks were both 30-day mortality, while the source and target domains were eCritical and MIMIC-III, respectively. AKI: acute kidney injury; DA: domain adaptation; ITL: inductive transfer learning; MIMIC-III: Medical Information Mart for Intensive Care III.

Transfer learning (TL) can be useful in these challenging scenarios. TL research using EHR data has been uncommon compared to medical image analysis and natural language processing. The basic idea of TL is to use the knowledge and representations learned while training the model on a source prediction task, and to improve prediction performance on a different, but potentially closely related target prediction task [2]. In practice, this is achieved by pretraining a model with data for the source prediction task and saving the trained weights. These weights capture the intrinsic knowledge of the data. This pretrained model is typically loaded without the final layers (usually the fully connected layers immediately preceding the output layer and the output layer itself), which are then replaced with new layers. Finally, this pretrained model is retrained to fine-tune it to the target prediction task (Figure 1).

In this work, we considered 2 commonly used types of TL methods. First, inductive transfer learning (ITL) aims to improve performance on the target task after learning a different but related source task, usually from the same domain [3]. For example, in the study by Tokuoka et al [4], a model trained for brain tissue annotation label (source task) was adapted to the task of brain tumor segmentation (target task) using magnetic resonance imaging images. Second, transductive TL, also referred to as domain adaptation (DA), makes use of different domains but the same prediction task [3]. For example, in the study by Titoriya and Sachdeva [5], AlexNet [6] model, which was pretrained on ImageNet data (source data set) for object classification (source task), was retrained on the Breakhis data set (target data set) to classify medical images into malignant or benign (target task) to predict breast cancer.

TL can be useful in a data-scarce scenario where the target data set does not have a sufficient volume to train a deep learning model but there is a sufficiently large source data set to train a pretrained model or a relevant pretrained model is available. For example, DA can be useful in a scenario where a rural intensive care unit (ICU) does not have sufficient data to train a model to predict a patient outcome, but an urban teaching hospital has a large data set to train a model for the same patient outcome. ITL can be useful when predicting new or rare patient outcomes at an ICU if that ICU has a sufficiently large data set for training a model to predict a different patient outcome.

TL research in medical image analysis is relatively comprehensive, as pretrained convolutional neural networks, such as AlexNet [6], ResNet [7], VGGNet [8], and GoogleNet [9], are publicly available and have been used widely for prediction problems such as image classification [10], image segmentation [11], object identification [12], disease categorization [13], and severity grading [14]. Most of these examples used DA rather than ITL.

Another field with significant previous TL research is natural language processing. Some of the established pretrained language models are Word2Vec [15], GloVe [16], Bidirectional Encoder Representations from Transformers [17], and fastText [18]. Some of the use cases of pretrained natural language processing models include text mining [19], word classification [20], and sentiment classification [21].

A recent trend has been to adapt the progress made in TL research in the natural language processing domain to EHR data analysis. Inspired by the pretrained Bidirectional Encoder Representations from Transformers model [17], Li et al [22] developed BEHRT using EHR data to improve future visit diagnosis prediction. This study used only 4 features, which included age, segment, position, and diagnosis. Longitudinal data were used, and a transformer-based model was trained. This study had inclusion criteria of patients with at least 5 visits and a diagnosis available in their EHR. One of the shortcomings of this study was not using all the available features in the EHR data such as demographics, laboratory results, vitals, and prescriptions. Another concern was the requirement of 5 previous visits with diagnosis, causing the model to be unable to predict the diagnosis for patients with fewer previous visits.

Liu et al [23] used TL to improve the prediction of acute kidney injury (AKI) using EHR data acquired at the University of Kansas Medical Center. Logistic regression (LR) was used as the global (baseline) model and the global TL model. Global TL and baseline models are the same LR models except the baseline model was trained on the original data set and the TL model was trained on the modified data set. To create the modified data set, data of each feature were multiplied by the corresponding feature coefficient, which was obtained from the global baseline model. The personalized model was then trained on a subset of the training sample with the highest similarity (nearest neighbors) with the selected test sample. For each test sample, a subset of the training data set with the highest k-nearest neighbor score was selected and a personalized model and personalized TL model were trained. The personalized TL model followed the same approach as the global TL model except that the training sample was selected from the modified data set using the k-nearest neighbor score (similarity) with the selected test sample. Although the source and target prediction tasks and domains were the same, this can be considered as DA since TL models use a modified data set. Here, deep learning models were not used, which are often assumed to be better at learning the general representations of the data. Since both these TL models (global and personalized) were not like the traditional pretrained models with saved weights, the transfer of knowledge happened by modifying the data set. Thus, the transferred knowledge is stored in a modified data set, not in the TL models. These TL models are strongly tied to this specific data set and their generalizability has not been well established by using external validation data.

In the study by Shickel et al [24], TL was used to improve hospital discharge prediction using a conventional ICU cohort acquired at the University of Florida Health as the source cohort and the intelligent ICU cohort also acquired at the University of Florida Health as the target cohort. It used a feed-forward neural network for TL. Here, the source cohort had 48,400 patient records whereas the target cohort had 51 patient records. This study used only 9 features and DA, even though not stated specifically in the paper. This semantic progression was observed in other studies as well; DA has become so prevalent that the terms TL and DA are being used interchangeably, with the former most commonly referring to the latter [24,25].

To the best of our knowledge, TL research has been rare in EHR-based ICU patient outcome prediction, particularly in terms of ITL, leading to a limited understanding of the effectiveness of TL in data-scarce ICU settings. Furthermore, there is a lack of publicly available EHR-based pretrained models for ICU patient outcome prediction. These are important gaps because EHR-based tabular data are one of the most widely used forms of data in predictive modeling studies in health, and the state-of-the-art TL methods from general computer vision and natural language processing are often not readily applicable to EHR data. Hence, this retrospective study aimed to compare the performances of DA, ITL, and baseline (without TL) models in predicting the following 4 ICU patient outcomes at varying levels of data scarcity: 30-day post-ICU admission mortality, AKI, hospital length of stay (H_LOS), and ICU length of stay (ICU_LOS).

## Methods

### Data Sources

EHR data from 2 patient cohorts were used in this retrospective study. The first cohort was eCritical, which has 55,689 unique admission records from 48,672 unique patients admitted to 15 ICUs in Alberta, Canada, between March 2013 and December 2019. The second cohort was the Medical Information Mart for Intensive Care III (MIMIC-III) database [26] version 1.4, which includes 61,532 unique admission records from 46,476 unique patients admitted to the ICUs at the Beth Israel Deaconess Medical Center in Boston, Massachusetts, between 2001 and 2012.

### Ethical Considerations

Since MIMIC-III is a publicly available database, the need to obtain research ethics approval to use it in this study was waived. However, eCritical contains patient identifying information, and approval from the Conjoint Health Research Ethics Board, University of Calgary, was obtained (REB17-0389). Informed consent was waived due to the large number of patients involved in the study. All research was performed in accordance with relevant guidelines and regulations set by the University of Calgary and Alberta Health Services, the custodian of the eCritical data, as well as the Declaration of Helsinki.

### Patient Cohorts

Two different sets of inclusion and exclusion criteria were applied. The first set was used to establish the base cohorts for the entire study, whereas the second set was specific to each patient outcome and applied to the base cohorts.

**Figure 2:**
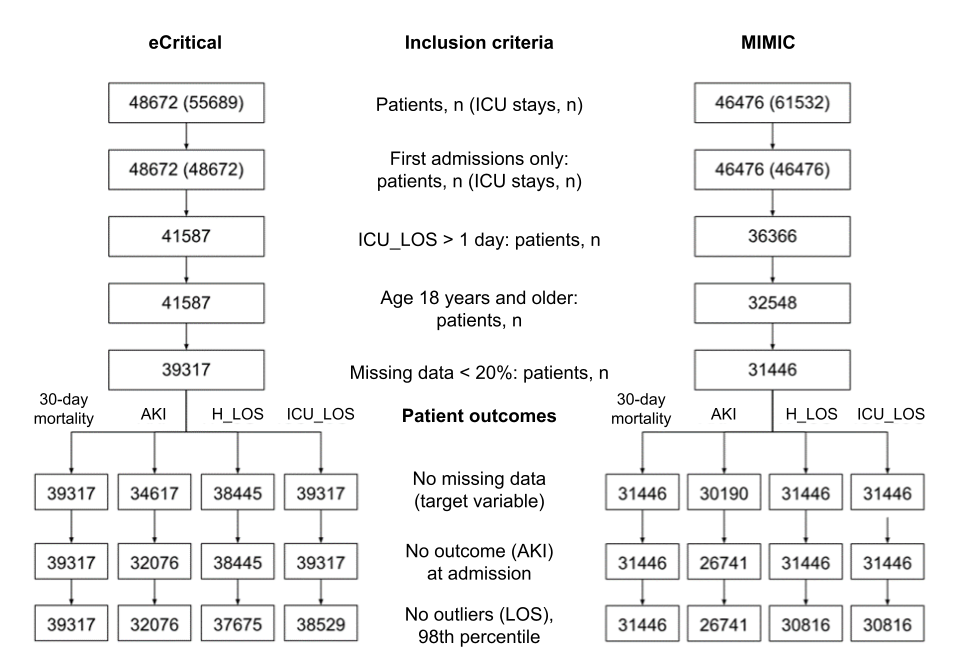
Patient cohort flowchart. AKI: acute kidney injury; H_LOS: hospital length of stay; ICU: intensive care unit; ICU_LOS: ICU length of stay; MIMIC: Medical Information Mart for Intensive Care.

For the base cohorts, the following inclusion criteria were applied to both eCritical and MIMIC-III (Figure 2): (1) only the first ICU admission of each patient; (2) ICU_LOS greater than 24 hours; (3) only adult patients with an age 18 years and older; and (4) samples with data available of at least 80% of the features (missing values are imputed as discussed in data preprocessing section). As a result, the base cohorts for eCritical and MIMIC-III consisted of 39,317 and 31,446 patient records, respectively.

Further inclusion and exclusion criteria were applied to each patient outcome, resulting in different data sets for each outcome, as shown in Figure 2. The 30-day mortality did not require further inclusion criteria and was modeled using the base cohorts. For AKI, the following criteria were applied: (1) only patient records with sufficient data to determine the presence or absence of AKI, with one serum creatinine laboratory measurement within the first 24 hours of admission to be able to establish a baseline and another measurement after 24 hours of ICU admission; and (2) no AKI onset at or within 24 hours of admission. For ICU_LOS, the following inclusion criteria were applied: (1) the presence of ICU admission and discharge date times; and (2) to exclude outliers only the bottom 98th percentile values of ICU_LOS are included. For H_LOS, the following inclusion criteria were applied: (1) the presence of hospital admission and discharge date times; and (2) to exclude outliers only the bottom 98th percentile values of H_LOS were included.

In the end, the eCritical and MIMIC-III cohorts had 39,317 and 31,446 samples, respectively, for 30-day mortality. Similarly, AKI had 32,076 and 26,741 samples, H_LOS had 37,675 and 30,816 samples, and ICU_LOS had 38,529 and 30,816 samples, respectively. These cohorts were randomly split into 80% training, 10% validation, and 10% test data. We compared the prediction performance of TL with those of baseline models at different levels of training data scarcity with random subsets of 1%, 5%, 10%, 25%, 50%, 75%, and 100% of the training data.

### Patient Outcomes

The primary patient outcomes for this study were 30-day post-ICU admission mortality and ICU_LOS. A patient was defined as deceased if he or she died after being admitted to the ICU and within 30 days of ICU admission. ICU_LOS was defined as the time between ICU admission and discharge.

Furthermore, AKI after 24 hours of ICU admission and H_LOS were predicted as secondary patient outcomes. AKI was identified using the creatinine criteria of KDIGO [27]. H_LOS was defined as the time between hospital admission and discharge.

30-day mortality and AKI were predicted as classification problems, whereas H_LOS and ICU_LOS were predicted as regression problems.

### Feature Set

Machine learning (ML) models were trained with the following predictor variables from the first 24 hours in the ICU that were common in both eCritical and MIMIC-III: demographics, vitals, laboratory test results, Glasgow Coma Scale, prescriptions, dialysis, and mechanical ventilation. Features with more than 30% missing data were excluded from the study. Table 1 shows a complete list of the predictor variables. Four statistical features (5th percentile, 95th percentile, IQR, and median) were extracted from the longitudinal variables such as vitals, laboratory results, and Glasgow Coma Scale. The maximum and minimum values of laboratory assessments and vitals carry crucial information regarding the health condition of the patient, but to minimize the influence of outliers, the 5 percentile and 95 percentiles were used instead. Other predictor variables, such as prescriptions, dialysis, and mechanical ventilation, were transformed into binary features to indicate the presence or absence. In the end, a total of 104 features were included in this study.

**Table 1:** Common feature set between eCritical and MIMIC-III^a^.

Feature	Category	Unit of measurement
Age	Demographics	years
Weight	Demographics	kg
Sex	Demographics	Binary (M/F)
Eye-opening	GCS^b^	N/A^c^
Verbal response	GCS	N/A
GCS	GCS	N/A
Motor response	GCS	N/A
Urine volumes	Urine volumes	mL
Heart rate	Vitals	bpm
BP^d^ systolic	Vitals	mm Hg
BP diastolic	Vitals	mm Hg
SpO_2_^e^	Vitals	%
Respiratory rate	Vitals	breaths/min
Urea blood	Laboratory findings	mmol/L
CO_2_ content blood	Laboratory findings	mmol/L
Creatinine blood	Laboratory findings	µmol/L
Glucose blood	Laboratory findings	mmol/L
Potassium blood	Laboratory findings	mmol/L
Sodium blood	Laboratory findings	mmol/L
pCO_2_^f^ arterial	Laboratory findings	mmHg
FiO_2_	Laboratory findings	%
PH arterial	Laboratory findings	N/A
pO_2_^g^ arterial	Laboratory findings	mmHg
Hemoglobin	Laboratory findings	g/L
Hematocrit	Laboratory findings	%
RBC^h^	Laboratory findings	E+12 units/L
WBC^i^	Laboratory findings	E+9 units/L
Dialysis	Dialysis	Binary (1/0)
Mechanical ventilation	Mechanical ventilation	Binary (1/0)
Norepinephrine	Prescriptions	Binary (1/0)
Phenylephrine	Prescriptions	Binary (1/0)
Vasopressin	Prescriptions	Binary (1/0)
Dobutamine	Prescriptions	Binary (1/0)
Dopamine	Prescriptions	Binary (1/0)
Epinephrine	Prescriptions	Binary (1/0)

^a^MIMIC-III: Medical Information Mart for Intensive Care III.

^b^GCS: Glasgow Coma Scale.

^c^N/A: not applicable.

^d^BP: blood pressure.

^e^SpO_2_: oxygen saturation.

^f^pCO_2_: partial pressure of carbon dioxide.

^g^pO_2_: partial pressure of oxygen.

^h^RBC: red blood cell.

^i^WBC: white blood cell.

### Data Preprocessing

Differences in units of measurement between eCritical and MIMIC-III were handled by converting all features in MIMIC-III to the eCritical units of measurement.

The train set was used for training the models, whereas the validation set was used for tuning hyperparameters. The test set was used for model performance evaluation. Numerical features (eg, vitals and laboratory findings) were scaled (unit variance and zero mean) and categorical features (eg, sex, prescriptions, and mechanical ventilation) were transformed using one-hot encoding.

Missing data were present to varying degrees in both cohorts. Features with more than 30% missing data were excluded from the study. Patient records with missing data for more than 20% of the features were dropped. The remaining patient records with missing values were imputed using the IterativeImputer from the Scikit-learn Python package, which is similar to the multiple imputation by chained equations. Imputation was performed after splitting the data to avoid data leakage. Training, validation, and test data were imputed separately.

Both categorical patient outcomes, 30-day mortality and AKI, had varying degrees of class imbalance in both cohorts. The 30-day mortality had the highest class imbalance; the event rates were 16.79% and 12.24% in eCritical and MIMIC-III, respectively. The class imbalance was mitigated using the Synthetic Minority Oversampling Technique [28], by oversampling the minority class to 50% of the majority class and then undersampling the majority class to 100% of the minority class.

### Baseline Models

Since logistic [29] and lasso [30] regression are widely used in medical research for classification and regression, respectively, they were used as baseline models. In addition, deep learning models (fully connected neural network [FCNN]) with random initialization of weights were used as baseline models.

Hyperparameter tuning for the LR and lasso models was done using grid search with 3-fold cross-validation. The searched hyperparameter space for LR included: solvers of newton-cg, liblinear, lbfgs, sag, and saga; penalties of L1, L2, and none; and C values ranging from 0.01 to 10 with a step of 0.01. The searched hyperparameter space for lasso included: α values ranging from .01 to 1 with a step of .01. FCNN baseline models used the same architecture and hyperparameters as the corresponding TL models so that we were comparing models that were trained the same way except how the weights were initialized.

Eight FCNN models were created for the 4 patient outcomes and 2 cohorts. Four LR models were created for 30-day mortality and AKI trained on eCritical and MIMIC-III. Similarly, 4 lasso regression models were created for H_LOS and ICU_LOS trained on eCritical and MIMIC-III.

### TL Models

In DA, the source and target domains were eCritical and MIMIC-III, respectively. The source and target tasks were the same, and each DA model predicted one of the four patient outcomes. Each model was pretrained on the source domain data before being fine-tuned and evaluated on the target domain data. As a result, four pretrained DA models were created, one for each of the 4 patient outcomes.

In ITL, both the source and target domains were eCritical and the source task was 30-day mortality prediction whereas the target task was the prediction of one of the four patient outcomes. The ITL model where both the source and target tasks were 30-day mortality served as a benchmark for the other ITL models. In the end, 4 pretrained ITL models were created for each of the 4 patient outcomes.

The pretrained TL models were FCNNs trained on the training data set of the source domain. Hyperparameters were tuned using the validation data set. The searched hyperparameter space included: dropout rates of 0.5, 0.4, and 0.3; batch sizes of 32, 64, and 128; numbers of neurons per hidden layer of 100, 128, 256, and 200; learning rates of 0.001 and 0.0001; activation functions of ReLU, tanh, selu, elu, LeakyReLU, and PReLU; kernel initializers of He Uniform and He Normal; and kernel regularizers of L2 (l2=1e-3) and L1 (l1=0.001). In addition, different architectures were explored. The first one had 3 hidden layers with layer, layer/2, and layer/4 number of neurons. The second architecture had 7 hidden layers with layer, layer*2, layer*2, layer, layer, layer/2, and layer/4 neurons. Here, the layer had 100, 200, 128, and 256 neurons. Finally, these models were tested using the hold-out test set from the source domain to identify the best-performing model concerning balanced accuracy (to account for class imbalance) for classification tasks and mean absolute error (MAE) for regression tasks. Then, these best-performing models were used as the pretrained models.

For fine-tuning, the pretrained model was loaded and the last hidden layer was replaced with a new hidden layer with randomly initialized weights. Then, all pretrained model layers were frozen (preventing those layers from learning) except for the newly added hidden layer and the model was trained to allow the new hidden layer to adjust its weights. Then, all layers were unfrozen (allowing weights to update) and the model was trained for the final time.

### Prediction Performance Comparisons at Varying Levels of Data Scarcity

To investigate prediction performance at varying levels of data scarcity, random subsets of 1%, 5%, 10%, 25%, 50%, and 75% were created from the full training data set (100%). To avoid selection bias [31], each subset was obtained 10 different times using 10 different random states. Models were trained on these 10 data subsets and then performance metrics from all models were then aggregated for each subset. As there were 6 subsets (1%, 5%, 10%, 25%, 50%, and 75%) and the full training data set of 100%, 61 (6×10+1) models were trained for each outcome and each model. For example, for AKI, 61 LR, 61 FCNN, 61 ITL, and 61 DA models were trained.

To obtain the median and 95% CI of the performance metrics, 1000 bootstrap samples of the test set were obtained for the full data set (100%), and for the random subsets, 100 bootstrap samples for each of the 10 random states (1000 in total) were created and then tested using these bootstrapped test sets.

All classification models were assessed using balanced accuracy as the primary metric (to account for class imbalance) and the following 4 secondary metrics on the hold-out test set: area under the receiver operating characteristic curve, accuracy, precision (also known as positive predictive value), and recall (also known as sensitivity). All regression models were evaluated using MAE and mean squared error (MSE).

Finally, Wilcoxon rank sum tests were performed to compare the performance of TL models to the baseline models. Since there were repeated comparisons involved, a Bonferroni correction was applied. Because the classification tasks had 35 comparisons (7 data subsets and 5 metrics), statistical significance was indicated by *P*<.001 (.05/35). The regression tasks had 14 comparisons (7 data subsets and 2 metrics), leading to statistical significance set at *P*<.001 (.05/14).

## Results

### Patient Cohorts

Based on the inclusion and exclusion criteria, the final eCritical and MIMIC-III cohorts were different for each patient outcome (Figure 2). The 30-day mortality cohort had 39,317 and 31,446 samples in eCritical and MIMIC-III databases, respectively, whereas the AKI cohort had 32,076 and 26,741 samples, respectively. The H_LOS cohort had 37,675 and 30,816 samples, and the ICU_LOS cohort had 38,529 and 30,816 samples, respectively. In the eCritical cohort, there were 6713 (17.07%) 30-day mortalities, whereas, in the MIMIC-III database, there were 3900 (12.40%) 30-day mortalities. The eCritical cohort had 4524 (14.11%) AKI cases whereas MIMIC-III had 5789 (21.64%) AKI cases. The eCritical cohort had a median H_LOS of 11.48 (IQR 5.59-23.29) days, whereas the MIMIC-III cohort had a median (IQR) of 7.39 (4.67-12.32). Similarly, the median (IQR) ICU_LOS in the eCritical and MIMIC-III cohorts were 3.97 (2.2-7.67) and 2.47 (1.59-4.58) days, respectively. The descriptive statistics for the 2 cohorts are shown in Table 2.

**Table 2:** Descriptive statistics of the 2 patient cohorts.

Descriptor	eCritical	MIMIC-III^a^
Male, n (%)	22,957 (58.39)	17,900 (56.92)
Age (years), median (IQR)	60 (46-70)	66 (53-78)
Admission weight (kg), median (IQR)	80 (67.4-96.6)	79.3 (66.5-94)
30-day mortality, n (%)	6713 (17.07)	3900 (12.40)
AKI^b^, n (%)	4524 (14.10)	5789 (21.64)
H_LOS^c^ (days), median (IQR)	11.48 (5.59-23.29)	7.39 (4.67-12.32)
ICU_LOS^d^ (days), median (IQR)	3.97 (2.2-7.67)	2.47 (1.59-4.58)
Blood creatinine (μmol/L), median (IQR)	90.5 (65.5-152.05)	79.56 (61.88-123.76)
Glasgow Coma Scale, median (IQR)	11 (7.2-14.25)	12 (8-15)
Blood glucose (mmol/L), median (IQR)	7.49 (6.09-9.45)	7.1 (5.83-8.99)
Blood potassium (mmol/L), median (IQR)	3.95 (3.6-4.4)	4.07 (3.7-4.5)
Blood sodium (mmol/L), median (IQR)	138.2 (135.15-141)	138.5 (136-141)
Arterial pH, median (IQR)	7.38 (7.32-7.43)	7.38 (7.33-7.43)
WBC^e^ (/µL), median (IQR)	12.03 (8.55-16.65)	11.2 (8.1-14.9)
RBC^f^ (/µL), median (IQR)	3.7 (3.15-4.24)	3.54 (3.15-3.99)
Systolic blood pressure (mm Hg), median (IQR)	118.5 (100.25-139.8)	117 (100.8-135.75)
Diastolic blood pressure (mm Hg), median (IQR)	61.5 (52-73.8)	59.5 (49.5-71)
SpO_2_^g^ (%), median (IQR)	97 (94-99)	97.7 (95-99.7)
Hemoglobin (g/L), median (IQR)	112.45 (95.75-129.2)	107 (94.5-121)
Hematocrit (%), median (IQR)	34 (29-39)	31.5 (27.95-35.5)
Mechanical ventilation, n (%)	34,073 (86.66)	16,429 (52.25)
Dialysis, n (%)	2173 (5.53)	6909 (21.97)
Norepinephrine, n (%)	15,797 (40.18)	3,339 (10.62)
Phenylephrine, n (%)	4384 (11.15)	6737 (21.42)
Vasopressin, n (%)	5087 (12.94)	712 (2.26)
Dobutamine, n (%)	754 (1.92)	429 (1.36)
Dopamine, n (%)	1051 (2.67)	1512 (4.81)
Epinephrine, n (%)	1320 (3.36)	1221 (3.88)

^a^MIMIC-III: Medical Information Mart for Intensive Care III.

^b^AKI: acute kidney injury.

^c^H_LOS: hospital length of stay.

^d^ICU_LOS: intensive care unit length of stay.

^e^WBC: white blood cell.

^f^RBC: red blood cell.

^g^SpO_2_: oxygen saturation.

### Pretrained Models

After hyperparameter tuning for the 30-day mortality source task, the pretrained model with 3 hidden layers of 128, 64, and 32 neurons was selected. This model had the highest balanced accuracy of 0.7810. This was the pretrained model for all 4 ITL target tasks and the 30-day mortality DA target task. Similarly, after hyperparameter tuning for the AKI, H_LOS, and ICU_LOS source tasks, pretrained models with 3 hidden layers of 256, 128, and 64 neurons, 7 hidden layers of 256, 512, 512, 256, 256, 128, and 64 neurons, and 7 hidden layers of 256, 512, 512, 256, 256, 128, and 64 neurons, with a balanced accuracy of 0.7199, an MAE of 11.8019, and an MAE of 3.0887, were selected, respectively. These were the pretrained models for the DA target tasks for AKI, H_LOS, and ICU_LOS, respectively.

These pretrained models are publicly available via GitHub [32].

### Domain Adaptation

**Figure 3:**
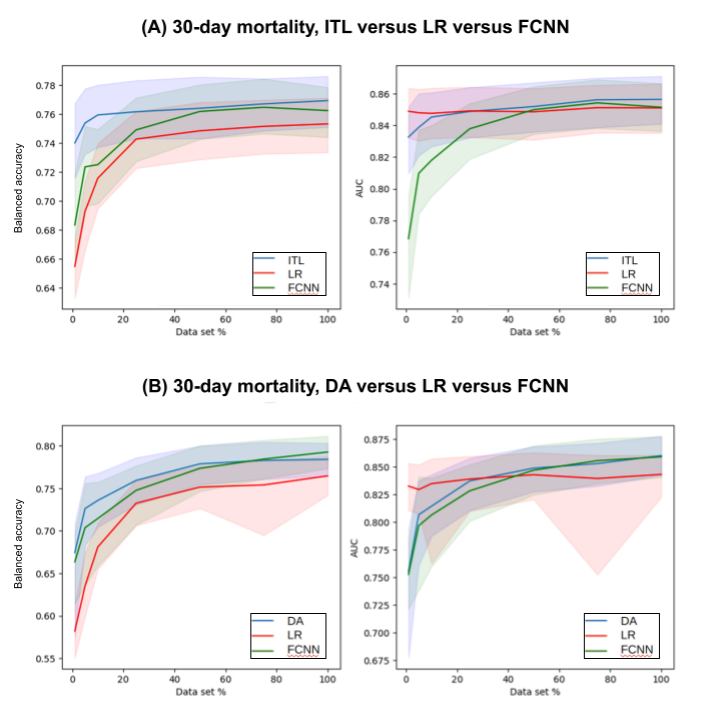
30-day mortality prediction performances of the (A) ITL and (B) DA models in comparison with those of the baseline models across a range of data subsets representing varying levels of data scarcity. The solid lines are the medians and the shaded areas are the 95% CIs. AUC: area under the receiver operating characteristic curve; DA: domain adaptation; FCNN: fully connected neural network; ITL: inductive transfer learning; LR: logistic regression.

**Figure 4:**
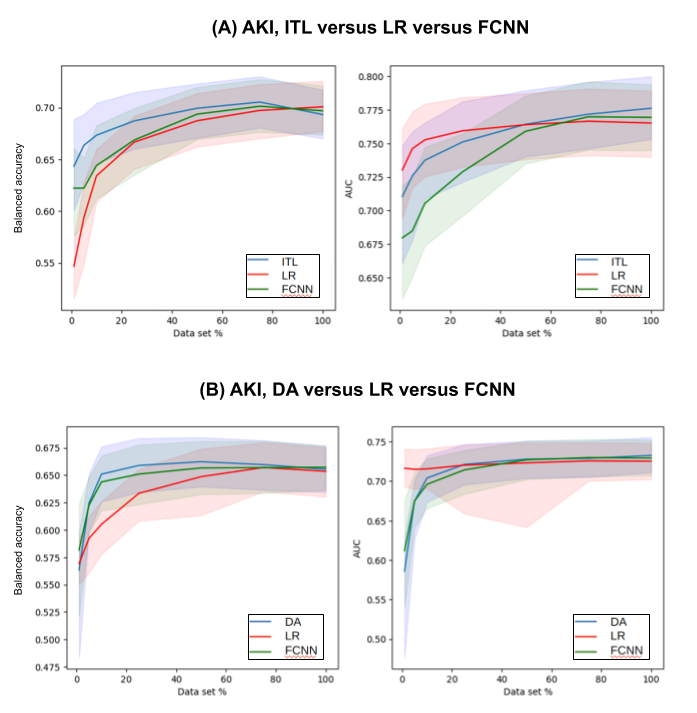
AKI prediction performances of the (A) ITL and (B) DA models in comparison with those of the baseline models across a range of data subsets representing varying levels of data scarcity. The solid lines are the medians and the shaded areas are the 95% CIs. AKI: acute kidney injury; AUC: area under the receiver operating characteristic curve; DA: domain adaptation; FCNN: fully connected neural network; ITL: inductive transfer learning; LR: logistic regression.

**Figure 5:**
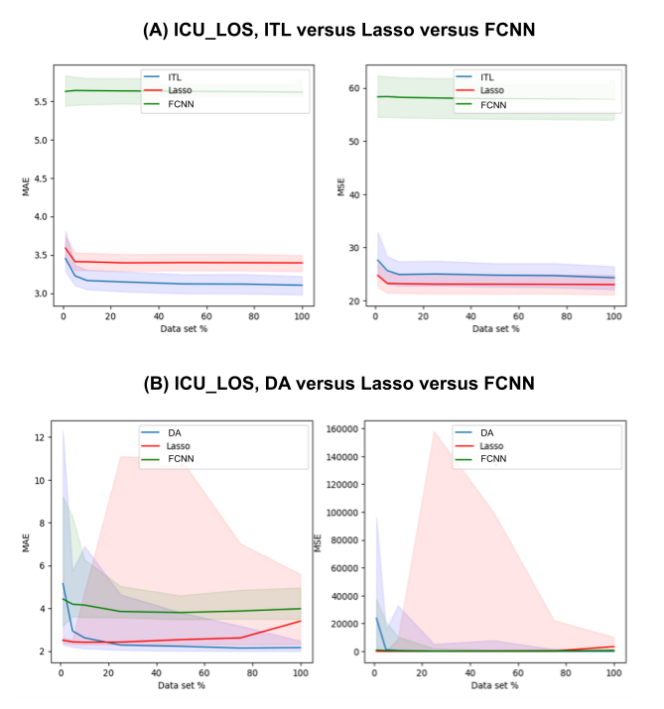
ICU_LOS prediction performances of the (A) ITL and (B) DA models in comparison with those of the baseline models across a range of data subsets representing varying levels of data scarcity. The solid lines are the medians and the shaded areas are the 95% CIs. DA: domain adaptation; FCNN: fully connected neural network; ICU_LOS: intensive care unit length of stay; ITL: inductive transfer learning; MAE: mean absolute error; MSE: mean squared error.

**Figure 6:**
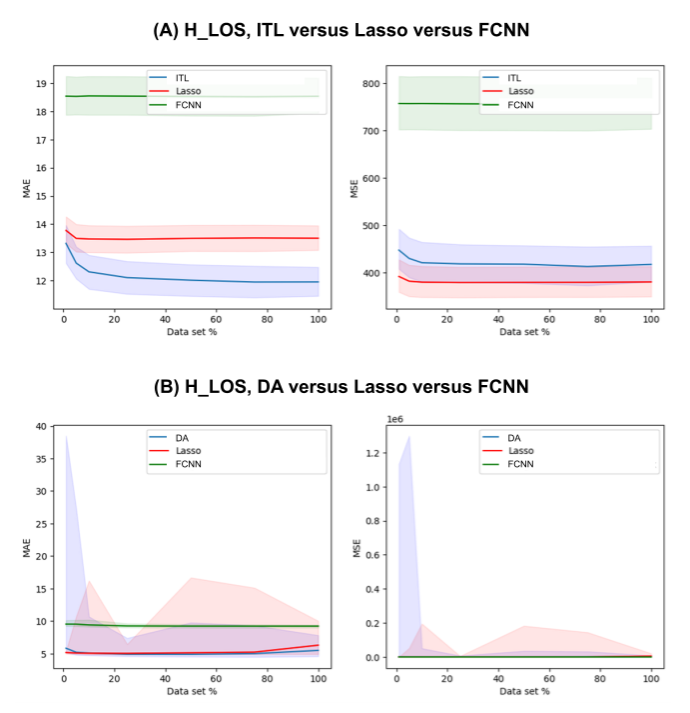
H_LOS prediction performances of the (A) ITL and (B) DA models in comparison with those of the baseline models across a range of data subsets representing varying levels of data scarcity. The solid lines are the medians and the shaded areas are the 95% CIs. DA: domain adaptation; FCNN: fully connected neural network; H_LOS: hospital length of stay; ITL: inductive transfer learning; MAE: mean absolute error; MSE: mean squared error.

Multimedia Appendices 1-4 show the complete prediction performances of the DA and baseline models at varying levels of data scarcity represented by the data subsets for 30-day mortality, AKI, ICU_LOS, and H_LOS, respectively. Figures 3-6 pictorially compare the DA and baseline models for each patient outcome.

For 30-day mortality, DA models outperformed both the baseline models LR and FCNN for data subsets 1% to 50%. For data sets, 75% and 100% DA models outperformed the LR model but underperformed the FCNN model. For example, when 1% data set was used for training, the DA model had a median balanced accuracy of 0.6744 (95% CI 0.5758-0.7083), whereas LR had 0.5821 (95% CI 0.551-0.6134) and FCNN had 0.6636 (95% CI 0.6146-0.6971).

For AKI, the DA models outperformed both baseline models for some of the data subsets (75%, 50%, 25%, 10%, and 5%) and underperformed both baseline models for the data subset (1%). The DA model outperformed the LR model and underperformed the FCNN model for the 100% data set. When the 10% data subset was used, the DA model had a median balanced accuracy of 0.6511 (95% CI 0.626-0.6763), whereas the LR model had 0.6052 (95% CI 0.5779-0.6262) and the FCNN model had 0.6439 (95% CI 0.6177-0.6678).

For ICU_LOS, the DA models outperformed both baseline models for some of the data subsets (25%-100%). The DA models outperformed the FCNN models but underperformed the lasso models in some cases (5% and 10%). In addition, the results from the 1% data subset were not significant between DA and FCNN (*P*=.05). When the 25% training data subset was used for training, the DA model had a median MAE of 2.2781 (95% CI 2.0427-4.643), whereas the lasso model had 2.4165 (95% CI 2.2967-11.1001) and the FCNN model had 3.8481 (95% CI 3.5641-5.0331).

For H_LOS, the DA models outperformed both baseline models for some of the data subsets (25%-100%). The DA models outperformed the FCNN models but underperformed the lasso models in some cases (1% and 5%). The results from the 10% data subset were not significantly different between DA and lasso (P*P*.02), but the DA model outperformed the FCNN model. When the 25% data subset was used for training, the DA model had a median MAE of 4.9109 (95% CI 4.5982-7.389), whereas the lasso model had 5.0491 (95% CI 4.8903-6.457) and the FCNN model had 9.2677 (95% CI 9.0162-9.6332).

### Inductive Transfer Learning

Multimedia Appendices 3-6 show the complete prediction performances of the ITL and baseline models at varying levels of data scarcity represented by the data subsets for 30-day mortality, AKI, ICU_LOS, and H_LOS, respectively. Figures 3-6 pictorially compare the ITL and baseline models for each patient outcome.

As mentioned in the Methods section, the 30-day mortality prediction results from ITL presented in Multimedia Appendix 5 serve as a benchmark, since the source and target domains and prediction tasks were the same. The fast convergence of ITL performance at very small data subsets shown in Figure 3 corroborates the limited learning taking place during fine-tuning.

For AKI, the ITL models outperformed both baseline models for all data subsets except 100%. For example, when the 1% data set was used for training the models, the ITL model had a median balanced accuracy of 0.6434 (95% CI 0.6006-0.6888), whereas the LR model had 0.5467 (95% CI 0.5154-0.5732) and the FCNN model had 0.6222 (95% CI 0.5757-0.6604).

For ICU_LOS, the ITL models outperformed both baseline models for all the data subsets. For example, when the 1% data set was used, the ITL model had a median MAE of 3.4519 (95% CI 3.2863-3.8158), whereas lasso had 3.5883 (95% CI 3.4255-3.7376) and FCNN had 5.626 (95% CI 5.4351-5.8329).

For H_LOS, the ITL models outperformed both baseline models for all data subsets. For example, when the 1% data set was used, the ITL model had a median MAE of 13.3182 (95% CI 12.6128-13.9609) whereas lasso had 13.7765 (95% CI 13.3118-14.2661) and FCNN had 18.5363 (95% CI 17.8711-19.243).

## Discussion

### Principal Results

Overall, the ITL models outperformed the baseline models in 55 of the 56 cases (7 data subsets × 4 outcomes × 2 baseline models). The DA models outperformed the baseline models 45 times in 56 cases. While TL is expected to yield better prediction performance than the baseline models, particularly with small data subsets, this assumption has not been confirmed in a comprehensive manner yet in the context of EHR-based ICU patient outcome prediction. In particular, the ITL prediction performances reported in this study are important contributions, given that ITL has seldom been investigated in EHR-based studies in general.

Moreover, the results from this study characterize DA, ITL, and baseline prediction performances as a function of target data volume. ICUs with limited data or computing resources can use our results as a guide to decide which would be better: fine-tune our pretrained models on their data or train new models from scratch using their small data set.

It is also worth noting that DA did not always outperform the baseline models even at very small data subsets (eg, ICU_LOS at the 1% subset; Multimedia Appendix 3). This finding implies that one should not blindly apply TL even when the target data set is small and expect performance improvement.

The ITL models performed better than the DA models in terms of the number of times and the margin with which they outperformed the baseline models. This speaks to the fact that the eCritical and MIMIC-III cohorts are quite different, and the knowledge learned from eCritical exhibited limited use in predicting the outcomes of the MIMIC-III patients. This is corroborated by the substantial differences in all 4 outcomes shown in Table 2. There seem to be more similarities between different outcomes within the same cohort than between different cohorts for the same outcome. This finding may be surprising to many researchers since the differences between different patient outcomes in terms of disease progression and risk factors are believed to be substantial, whereas even at different sites, the fundamentals of the diseases and ICU care should have many similarities.

Our pretrained models have been made available publicly which can be used at other ICUs or in future research. In many scenarios, TL was useful even at the 1% data subset, representing only about 200 samples. Hence, ICUs with their own data set containing just 200 samples can potentially benefit from our pretrained models. Given the paucity of public pretrained models in EHR-based ICU patient outcome prediction, our pretrained models are an invaluable contribution to the field.

### Clinical Implications

While accurate patient outcome prediction in the ICU has the potential to enable early initiation of preventative care and improve clinical efficiency and care resource management, it may be infeasible for many ICUs to build their own predictive models due to a lack of digital data infrastructure. The pretrained models from this study address this barrier by serving as predictive models that can be used out-of-the-box (albeit with suboptimal performance) or fine-tuned with a small amount of local data. This study provides a pathway for a wider group of ICUs to consider bringing patient outcome prediction models to the point of care.

### Comparisons With the Literature

This study has several strengths in comparison with previous EHR-based TL studies. First, this study is one of the few studies that explored ITL using EHR data. Second, this study used 2 large cohorts: eCritical with 55,689 ICU admissions from 48,672 patients as the source domain, and MIMIC-III with 61,532 ICU admissions from 46,476 patients as the target domain. These are considerably larger than the cohorts used in previous DA studies. For example, Shickel et al [24] used the conventional ICU cohort at the University of Florida Health with 48,400 distinct ICU admissions as the source domain and the intelligent ICU cohort at the University of Florida Health with only 51 ICU admissions as the target cohort. Third, this study used a large feature set of 104. In comparison, Shickel et al [24] used only 9 features, while Li et al [22] used 4 features. Fourth, our pretrained models are able to predict outcomes for new patients without previous admissions, unlike the natural language processing models developed by Li et al [22].

### Secondary Performance Metric Results

While the TL models outperformed the baseline models in general with respect to the primary performance metrics (balanced accuracy and MAE), the baseline models (particularly LR and lasso) often outperformed the TL models in terms of the secondary metrics. In mortality and AKI prediction, the LR models tended to show higher precisions and lower recalls than the TL models. Given that both mortality and AKI exhibited low event rates leading to substantial class imbalance, the precision and recall results indicate that the LR models were more biased toward the majority class than the TL models. The higher area under the receiver operating characteristic curves from the LR models indicates that they achieved higher specificities in general than the TL models, further corroborating the bias toward the majority class. This is why we chose balanced accuracy as our primary metric since it reflects the performances of both the majority and minority classes.

In the regression tasks of ICU_LOS and H_LOS prediction, the lasso models often yielded better results than the TL models in terms of MSE. This implies that the TL models often led to larger errors that were amplified by the squaring effect of MSE. Similarly, the large confidence intervals in Figures 5B and 6B are also likely due to occasional large errors caused by the fact that the model output is not upper-bounded.

### Limitations

This study has limitations. First, we could not include all available features due to the constraint of having to use common features in both eCritical and MIMIC-III. Second, this study could not investigate other major ICU patient outcomes, such as sepsis, delirium, and acute respiratory distress syndrome, due to data unavailability in either or both eCritical and MIMIC-III. Third, only 2 baseline models were investigated per prediction task, and more advanced ML models (eg, XGBoost [XGBoost Contributors]) were not used. However, because the primary objective of this study was to demonstrate the benefits of TL, the most appropriate benchmark models were the FCNNs where everything was equivalent to the TL models except for the use of a pretrained model. While our focus was not to produce the best prediction performance, our results are comparable to the best MIMIC-based results in the literature as shown by the review study conducted by Syed et al [33]. Fourth, this study only examined the discrimination of the prediction models and did not investigate calibration. While many health ML studies focus only on discrimination and neglect calibration [34], this remains an important limitation of this study. Finally, even though some of the features, such as vitals and laboratory findings, were longitudinal, we performed a cross-sectional study via feature aggregation. More advanced recurrent deep learning models (eg, long short-term memory and gated recurrent unit) that can leverage longitudinal information may have led to different results.

### Future Work

First, future work can include an investigation of DA and ITL on other ICU patient outcomes, such as sepsis and delirium, using data sets that can support such research. Second, the application of DA and ITL to more advanced recurrent deep learning models would be worthwhile. Third, the effectiveness of pretrained models in the combination of both DA and ITL (ie, both the domain and prediction task would change from source to target) remains to be studied. For example, a mortality prediction model pretrained on a source data set can be fine-tuned on a target data set from a different domain to predict AKI. Finally, the effectiveness of TL across patient subgroups with respect to demographics and socioeconomic status would be worthwhile investigating.

### Conclusions

In this retrospective study, we found that TL can lead to improved prediction performance when compared to baseline models trained from scratch only using target data. This performance improvement was observed in a wide range of simulated data scarcity. In addition, the performance of ITL was superior to that of DA. This implies fine-tuning a pretrained model to predict a different patient outcome within the same domain would be a promising approach. We hope that the pretrained models from this study are useful to other researchers and ICUs.

## Appendix

Multimedia Appendix 1 Thirty-day mortality prediction performances of domain adaptation and baseline models across all data subsets.

Multimedia Appendix 2 Acute kidney injury prediction performances of domain adaptation and baseline models across all data subsets.

Multimedia Appendix 3 Intensive care unit length of stay prediction performances of inductive transfer learning, domain adaptation, and baseline models across all data subsets.

Multimedia Appendix 4 Hospital length of stay prediction performances of inductive transfer learning, domain adaptation, and baseline models across all data subsets.

Multimedia Appendix 5 Thirty-day mortality prediction performances of inductive transfer learning and baseline models across all data subsets.

Multimedia Appendix 6 Acute kidney injury prediction performances of inductive transfer learning and baseline models across all data subsets.

## Abbreviations

AKI: acute kidney injury
DA: domain adaptation
EHR: electronic health record
FCNN: fully connected neural network
H_LOS: hospital length of stay
ICU: intensive care unit
ICU_LOS: intensive care unit length of stay
ITL: inductive transfer learning
LR: logistic regression
MAE: mean absolute error
MIMIC-III: Medical Information Mart for Intensive Care III
ML: machine learning
MSE: mean squared error
TL: transfer learning

## Data Availability

The Medical Information Mart for Intensive Care (MIMIC) data sets analyzed during this study are available in the MIMIC-III repository [35]. The eCritical data sets analyzed during this study are not publicly available due to their patient-identifiable information but are available from the corresponding author upon reasonable request. The code that conducted the experiments, as well as the pretrained models, are available on GitHub [32].
