# Supplementary material for: Using Domain Adaptation and Inductive Transfer Learning to Improve Patient Outcome Prediction in the Intensive Care Unit: Retrospective Observational Study
Source: J Med Internet Res. 2024 Aug 21;26:e52730. doi: 10.2196/52730 (PMC11375375; doi:10.2196/52730)
Supplement: Multimedia Appendix 1 [file jmir_v26i1e52730_app1.docx]

P-values are from Wilcoxon rank sum tests. DA: domain adaptation; LR: logistic regression; FCNN: fully-connected neural network; AUC: area under the receiver operating characteristic curve.

| **Model** | **Data set %** | **Balanced Accuracy (95% CI)** | **p-value** | **AUC (95% CI)** | **p-value** | **Accuracy (95% CI)** | **p-value** | **Precision (95% CI)** | **p-value** | **Recall (95% CI)** | **p-value** |
| --- | --- | --- | --- | --- | --- | --- | --- | --- | --- | --- | --- |
| **DA** | 1% | 0.6744 (0.5758, 0.7083) |  | 0.7554 (0.6775, 0.7942) |  | 0.7835 (0.7510, 0.8130) |  | 0.2939 (0.2405, 0.3367) |  | 0.5333 (0.2670, 0.5956) |  |
| **LR** |  | 0.5821 (0.551, 0.6134) | <0.001 | 0.8326 (0.8105, 0.8533) | <0.001 | 0.8738 (0.8610, 0.8859) | <0.001 | 0.4987 (0.4166, 0.5886) | <0.001 | 0.1919 (0.1231, 0.2587) | <0.001 |
| **FCNN** |  | 0.6636 (0.6146, 0.6971) | <0.001 | 0.7527 (0.7214, 0.7827) | 0.2381 | 0.7990 (0.7790, 0.8210) | <0.001 | 0.3096 (0.2686, 0.3521) | <0.001 | 0.4853 (0.3500, 0.5627) | <0.001 |
| **DA** | 5% | 0.7262 (0.6826, 0.7639) |  | 0.8067 (0.7608, 0.8375) |  | 0.7730 (0.7443, 0.7978) |  | 0.3125 (0.2715, 0.355) |  | 0.6634 (0.5773, 0.7506) |  |
| **LR** |  | 0.6351 (0.5992, 0.6747) | <0.001 | 0.8295 (0.8074, 0.8519) | <0.001 | 0.8684 (0.8550, 0.8817) | <0.001 | 0.4707 (0.4113, 0.5512) | <0.001 | 0.3201 (0.2442, 0.4143) | <0.001 |
| **FCNN** |  | 0.7037 (0.6359, 0.7561) | <0.001 | 0.7968 (0.7382, 0.8410) | <0.001 | 0.8083 (0.7870, 0.8353) | <0.001 | 0.3426 (0.2907, 0.3969) | <0.001 | 0.5682 (0.4062, 0.6745) | <0.001 |
| **DA** | 10% | 0.7358 (0.7045, 0.7678) |  | 0.8144 (0.7874, 0.8430) |  | 0.7687 (0.7358, 0.7946) |  | 0.3126 (0.2749,0.3534) |  | 0.6911 (0.6188, 0.7867) |  |
| **LR** |  | 0.6811 (0.6549, 0.7092) | <0.001 | 0.8349 (0.7623, 0.8574) | <0.001 | 0.8525 (0.7927, 0.8674) | <0.001 | 0.4215 (0.2985, 0.4761) | <0.001 | 0.4570 (0.4036, 0.5245) | <0.001 |
| **FCNN** |  | 0.7145 (0.6584, 0.7573) | <0.001 | 0.8064 (0.7598, 0.8399) | <0.001 | 0.8076 (0.7701, 0.8321) | <0.001 | 0.3449 (0.2915, 0.3977) | <0.001 | 0.594 (0.4698, 0.6975) | <0.001 |
| **DA** | 25% | 0.7594 (0.7308, 0.7861) |  | 0.8373 (0.8105, 0.8579) |  | 0.7612 (0.7418, 0.7790) |  | 0.3153 (0.2836, 0.345) |  | 0.7570 (0.6985, 0.8147) |  |
| **LR** |  | 0.7325 (0.7064, 0.7597) | <0.001 | 0.8390 (0.8100, 0.8596) | <0.001 | 0.8216 (0.8022, 0.8378) | <0.001 | 0.3743 (0.3324, 0.4144) | <0.001 | 0.6135 (0.5654, 0.6657) | <0.001 |
| **FCNN** |  | 0.7477 (0.7068, 0.7765) | <0.001 | 0.8283 (0.8010, 0.8520) | <0.001 | 0.7790 (0.7456, 0.8010) | <0.001 | 0.3245 (0.2868, 0.3634) | <0.001 | 0.7153 (0.6034, 0.7687) | <0.001 |
| **DA** | 50% | 0.7788 (0.7543, 0.7999) |  | 0.8489 (0.8275, 0.8684) |  | 0.7606 (0.7266, 0.7863) |  | 0.3204 (0.2856, 0.3543) |  | 0.8041 (0.7437, 0.8561) |  |
| **LR** |  | 0.7515 (0.7265, 0.7763) | <0.001 | 0.8429 (0.8205, 0.8630) | <0.001 | 0.8010 (0.7809, 0.8175) | <0.001 | 0.3521 (0.3134, 0.3893) | <0.001 | 0.6855 (0.6366, 0.7340) | <0.001 |
| **FCNN** |  | 0.7736 (0.7465, 0.8004) | <0.001 | 0.8470 (0.8247, 0.8692) | <0.001 | 0.7650 (0.7157, 0.7860) | <0.001 | 0.3224 (0.2805, 0.3568) | 0.0099 | 0.7933 (0.7133, 0.8505) | <0.001 |
| **DA** | 75% | 0.7829 (0.7607, 0.8045) |  | 0.8530 (0.8326, 0.8716) |  | 0.7660 (0.7418, 0.7882) |  | 0.3268 (0.2949,0.3623) |  | 0.8052 (0.7564,0.8521) |  |
| **LR** |  | 0.7540 (0.6948, 0.7824) | <0.001 | 0.8395 (0.7524, 0.8606) | <0.001 | 0.7873 (0.7046, 0.8060) | <0.001 | 0.3362 (0.2495,0.376) | <0.001 | 0.7115 (0.6616,0.7630) | <0.001 |
| **FCNN** |  | 0.7844 (0.7612, 0.8068) | <0.001 | 0.8556 (0.8349, 0.8752) | <0.001 | 0.7599 (0.7316, 0.7784) | <0.001 | 0.3226 (0.2908,0.3525) | <0.001 | 0.8171 (0.7677,0.8815) | <0.001 |
| **DA** | 100% | 0.7840 (0.7645, 0.8033) |  | 0.8602 (0.8424, 0.8778) |  | 0.7488 (0.7326, 0.7644) |  | 0.3146 (0.2879,0.3433) |  | 0.8304 (0.7979,0.8647) |  |
| **LR** |  | 0.7647 (0.7417, 0.7873) | <0.001 | 0.8432 (0.8227, 0.8606) | <0.001 | 0.7819 (0.7676, 0.7968) | <0.001 | 0.3367 (0.305,0.3682) | <0.001 | 0.7410 (0.6982,0.7840) | <0.001 |
| **FCNN** |  | 0.7927 (0.7733, 0.8114) | <0.001 | 0.8591 (0.8405,0.8776) | <0.001 | 0.7396 (0.7224,0.7558) | <0.001 | 0.3104 (0.2855,0.339) | <0.001 | 0.8648 (0.8292,0.8965) | <0.001 |
