# Supplementary material for: Using Domain Adaptation and Inductive Transfer Learning to Improve Patient Outcome Prediction in the Intensive Care Unit: Retrospective Observational Study
Source: J Med Internet Res. 2024 Aug 21;26:e52730. doi: 10.2196/52730 (PMC11375375; doi:10.2196/52730)
Supplement: Multimedia Appendix 2 [file jmir_v26i1e52730_app2.docx]

P-values are from Wilcoxon rank sum tests. DA: domain adaptation; LR: logistic regression; FCNN: fully-connected neural network; AUC: area under the receiver operating characteristic curve.

| **Model** | **Data set %** | **Balanced Accuracy (95% CI)** | **p-value** | **AUC (95% CI)** | **p-value** | **Accuracy (95% CI)** | **p-value** | **Precision (95% CI)** | **p-value** | **Recall (95% CI)** | **p-value** |
| --- | --- | --- | --- | --- | --- | --- | --- | --- | --- | --- | --- |
| **DA** | 1% | 0.5635 (0.4834, 0.5981) |  | 0.5860 (0.4766, 0.6287) |  | 0.5757 (0.5249, 0.6318) |  | 0.2651 (0.1951, 0.3027) |  | 0.5366 (0.2346, 0.6283) |  |
| **LR** |  | 0.5693 (0.5502, 0.5917) | <0.001 | 0.7164 (0.6928, 0.7409) | <0.001 | 0.7925 (0.7776, 0.8075) | <0.001 | 0.5840 (0.5065, 0.6667) | <0.001 | 0.1726 (0.1294, 0.2263) | <0.001 |
| **FCNN** |  | 0.5817 (0.5212, 0.6242) | <0.001 | 0.6119 (0.5390, 0.6755) | <0.001 | 0.6385 (0.4097, 0.6991) | <0.001 | 0.2928 (0.2258, 0.3595) | <0.001 | 0.5059 (0.3051, 0.7725) | 0.1855 |
| **DA** | 5% | 0.6247 (0.5984, 0.6516) |  | 0.6746 (0.6271, 0.7020) |  | 0.6191 (0.5888, 0.6688) |  | 0.3166 (0.2848, 0.3560) |  | 0.6291 (0.5589, 0.6843) |  |
| **LR** |  | 0.5923 (0.5609, 0.6126) | <0.001 | 0.7150 (0.6899, 0.7399) | <0.001 | 0.7914 (0.7757, 0.8079) | <0.001 | 0.5561 (0.4865, 0.6320) | <0.001 | 0.2394 (0.1555, 0.2823) | <0.001 |
| **FCNN** |  | 0.6228 (0.5974, 0.6486) | <0.001 | 0.6748 (0.6415, 0.7114) | <0.001 | 0.6733 (0.6288, 0.7036) | <0.001 | 0.3405 (0.3052, 0.3795) | <0.001 | 0.5381 (0.4566, 0.6168) | <0.001 |
| **DA** | 10% | 0.6511 (0.626, 0.6763) |  | 0.7039 (0.6733, 0.7323) |  | 0.6533 (0.6288, 0.6796) |  | 0.3436 (0.3142, 0.3754) |  | 0.6498 (0.5881, 0.7007) |  |
| **LR** |  | 0.6052 (0.5779, 0.6262) | <0.001 | 0.7155 (0.6906, 0.7412) | <0.001 | 0.7865 (0.7675, 0.8049) | <0.001 | 0.5235 (0.4489, 0.6107) | <0.001 | 0.2851 (0.2007, 0.3361) | <0.001 |
| **FCNN** |  | 0.6439 (0.6177, 0.6678) | <0.001 | 0.6960 (0.6655, 0.7283) | <0.001 | 0.6656 (0.6378, 0.7189) | <0.001 | 0.3482 (0.3149, 0.4017) | <0.001 | 0.6008 (0.5142, 0.6584) | <0.001 |
| **DA** | 25% | 0.659 (0.6345, 0.6838) |  | 0.7212 (0.6954, 0.7466) |  | 0.6538 (0.6262, 0.6867) |  | 0.3475 (0.3176, 0.3829) |  | 0.6667 (0.6156, 0.715) |  |
| **LR** |  | 0.6335 (0.6083, 0.6557) | <0.001 | 0.7203 (0.6587, 0.7461) | <0.001 | 0.7630 (0.7159, 0.7821) | <0.001 | 0.4517 (0.3689, 0.5019) | <0.001 | 0.4079 (0.3628, 0.4612) | <0.001 |
| **FCNN** |  | 0.6511 (0.6234, 0.6779) | <0.001 | 0.7142 (0.6835, 0.739) | <0.001 | 0.6531 (0.6280, 0.6778) | <0.001 | 0.3430 (0.3102, 0.3768) | <0.001 | 0.6455 (0.5996, 0.6966) | <0.001 |
| **DA** | 50% | 0.6624 (0.6395, 0.6847) |  | 0.7278 (0.7038, 0.7508) |  | 0.6591 (0.6392, 0.6800) |  | 0.3527 (0.3233, 0.3814) |  | 0.6672 (0.6226, 0.7158) |  |
| **LR** |  | 0.6487 (0.6133, 0.6742) | <0.001 | 0.7231 (0.6416, 0.7484) | <0.001 | 0.7404 (0.6755, 0.7593) | <0.001 | 0.4181 (0.3362, 0.4599) | <0.001 | 0.4910 (0.4473, 0.5378) | <0.001 |
| **FCNN** |  | 0.6567 (0.6324, 0.6811) | <0.001 | 0.7270 (0.7023, 0.7512) | 0.1298 | 0.6538 (0.6314, 0.6826) | <0.001 | 0.3478 (0.3176, 0.3789) | <0.001 | 0.6593 (0.6104, 0.7161) | <0.001 |
| **DA** | 75% | 0.6597 (0.6366, 0.6819) |  | 0.7289 (0.7051, 0.7512) |  | 0.6535 (0.6314, 0.6759) |  | 0.3479 (0.3191, 0.3780) |  | 0.6710 (0.6243, 0.7119) |  |
| **LR** |  | 0.6571 (0.6352, 0.68) | <0.001 | 0.7256 (0.7004, 0.7488) | <0.001 | 0.7329 (0.7136, 0.7492) | <0.001 | 0.4110 (0.3740, 0.4455) | <0.001 | 0.5236 (0.4825, 0.5675) | <0.001 |
| **FCNN** |  | 0.657 (0.6336, 0.6811) | <0.001 | 0.7298 (0.7055, 0.7531) | <0.001 | 0.6542 (0.6321, 0.6759) | 0.5938 | 0.3470 (0.3175, 0.3768) | <0.001 | 0.6633 (0.6135, 0.7096) | <0.001 |
| **DA** | 100% | 0.6555 (0.6345, 0.6766) |  | 0.7326 (0.7111, 0.7554) |  | 0.6467 (0.6292, 0.6647) |  | 0.3430 (0.3152, 0.3703) |  | 0.6720 (0.6338, 0.7092) |  |
| **LR** |  | 0.6536 (0.6303, 0.6754) | <0.001 | 0.7253 (0.7021, 0.7483) | <0.001 | 0.7193 (0.7017, 0.7365) | <0.001 | 0.3959 (0.3596, 0.4323) | <0.001 | 0.5372 (0.4941, 0.5793) | <0.001 |
| **FCNN** |  | 0.6573 (0.6364, 0.6772) | <0.001 | 0.7292 (0.7073, 0.7522) | <0.001 | 0.6564 (0.6393, 0.674) | <0.001 | 0.3494 (0.3217, 0.3786) | <0.001 | 0.6584 (0.6208, 0.6954) | <0.001 |
