# Supplementary material for: Using Domain Adaptation and Inductive Transfer Learning to Improve Patient Outcome Prediction in the Intensive Care Unit: Retrospective Observational Study
Source: J Med Internet Res. 2024 Aug 21;26:e52730. doi: 10.2196/52730 (PMC11375375; doi:10.2196/52730)
Supplement: Multimedia Appendix 3 [file jmir_v26i1e52730_app3.docx]

P-values are from Wilcoxon rank sum tests. ITL: inductive transfer learning; DA: domain adaptation; FCNN: fully-connected neural network; MAE: mean absolute error; MSE: mean squared error.

| **Model** | **Data set %** | **MAE (95% CI)** | **p-value** | **MSE (95% CI)** | **p-value** |
| --- | --- | --- | --- | --- | --- |
| ITL | 1% | 3.4519 (3.2863, 3.8158) |  | 27.6137 (25.1995, 32.8881) |  |
| Lasso |  | 3.5883 (3.4255, 3.7376) | <0.001 | 24.7468 (22.5824, 26.9871) | <0.001 |
| FCNN |  | 5.626 (5.4351, 5.8329) | <0.001 | 58.3428 (54.539, 62.3479) | <0.001 |
| ITL | 5% | 3.227 (3.1007, 3.3694) |  | 25.6429 (23.1118, 28.3068) |  |
| Lasso |  | 3.4105 (3.2982, 3.5284) | <0.001 | 23.2357 (21.4593, 25.1419) | <0.001 |
| FCNN |  | 5.6384 (5.4493, 5.8144) | <0.001 | 58.3837 (54.5012, 62.1446) | <0.001 |
| ITL | 10% | 3.1657 (3.0505, 3.3016) |  | 24.8945 (22.7712, 27.3443) |  |
| Lasso |  | 3.4096 (3.3012, 3.5246) | <0.001 | 23.18 (21.4, 25.0781) | <0.001 |
| FCNN |  | 5.6367 (5.4538, 5.7984) | <0.001 | 58.2548 (54.4201, 62.0101) | <0.001 |
| ITL | 25% | 3.1476 (3.0217, 3.2813) |  | 25.0213 (22.786, 27.4814) |  |
| Lasso |  | 3.396 (3.2892, 3.5072) | <0.001 | 23.0958 (21.3047, 24.9569) | <0.001 |
| FCNN |  | 5.6324 (5.4662, 5.7974) | <0.001 | 58.1206 (54.3058, 61.8413) | <0.001 |
| ITL | 50% | 3.122 (2.9975, 3.2463) |  | 24.788 (22.6, 27.0035) |  |
| Lasso |  | 3.3986 (3.2954, 3.5105) | <0.001 | 23.085 (21.2642, 24.9566) | <0.001 |
| FCNN |  | 5.6285 (5.4505, 5.7924) | <0.001 | 57.9696 (54.1726, 61.8323) | <0.001 |
| ITL | 75% | 3.1191 (2.9939, 3.2486) |  | 24.6982 (22.4658, 27.0108) |  |
| Lasso |  | 3.3979 (3.2941, 3.5107) | <0.001 | 23.0554 (21.2866, 24.9292) | <0.001 |
| FCNN |  | 5.6235 (5.4485, 5.7827) | <0.001 | 57.9345 (54.1003, 61.6588) | <0.001 |
| ITL | 100% | 3.1047 (2.981, 3.2212) |  | 24.2975 (22.0683, 26.4235) |  |
| Lasso |  | 3.3957 (3.2838, 3.4947) | <0.001 | 23.0094 (21.1029, 24.7732) | <0.001 |
| FCNN |  | 5.6164 (5.4552, 5.784) | <0.001 | 57.7946 (53.9761, 61.3585) | <0.001 |
| DA | 1% | 5.151 (2.2976, 12.2704) |  | 23713.6811 (17.6564, 96207.6088) |  |
| Lasso |  | 2.5026 (2.3905, 2.6319) | <0.001 | 13.8609 (12.4635, 15.3404) | <0.001 |
| FCNN |  | 4.4312 (3.16, 9.2178) | 0.0468 | 857.9225 (27.3788, 37542.4647) | <0.001 |
| DA | 5% | 2.9333 (2.1891, 5.7482) |  | 1169.1479 (15.9935, 15706.9793) |  |
| Lasso |  | 2.4269 (2.3197, 2.5273) | <0.001 | 13.5643 (12.2516, 14.898) | <0.001 |
| FCNN |  | 4.1887 (3.6145, 8.2767) | <0.001 | 506.2204 (28.754, 21199.7618) | <0.001 |
| DA | 10% | 2.6185 (2.1133, 6.8996) |  | 587.236 (15.0171, 32741.8528) |  |
| Lasso |  | 2.4102 (2.3044, 4.7573) | <0.001 | 13.5523 (12.2645, 8800.3305) | <0.001 |
| FCNN |  | 4.1472 (3.5754, 6.272) | <0.001 | 301.9264 (28.5981, 10331.9659) | <0.001 |
| DA | 25% | 2.2781 (2.0427, 4.643) |  | 54.3868 (13.8313, 5248.2596) |  |
| Lasso |  | 2.4165 (2.2967, 11.1001) | <0.001 | 13.7606 (12.1696, 157997.5872) | <0.001 |
| FCNN |  | 3.8481 (3.5641, 5.0331) | <0.001 | 53.7051 (28.1006, 2100.1459) | 0.139 |
| DA | 50% | 2.2202 (2.0045, 3.8034) |  | 78.072 (13.2146, 7832.6985) |  |
| Lasso |  | 2.5322 (2.3006, 10.9919) | <0.001 | 43.2711 (12.4713, 99425.7167) | <0.001 |
| FCNN |  | 3.7991 (3.4774, 4.5941) | <0.001 | 171.9689 (27.1513, 1055.4228) | <0.001 |
| DA | 75% | 2.1394 (1.9897, 3.1683) |  | 22.771 (12.9367, 1438.1044) |  |
| Lasso |  | 2.6141 (2.2903, 7.0188) | <0.001 | 192.3652 (12.3675, 22244.873) | <0.001 |
| FCNN |  | 3.8703 (3.5031, 4.854) | <0.001 | 231.4307 (27.4148, 1779.207) | <0.001 |
| DA | 100% | 2.1608 (1.9881, 2.4793) |  | 55.7047 (12.5679, 142.0451) |  |
| Lasso |  | 3.3971 (2.3057, 5.5887) | <0.001 | 3402.6991 (12.7064, 10183.4661) | <0.001 |
| FCNN |  | 3.9776 (3.4847, 4.9573) | <0.001 | 588.9992 (26.9067, 1713.0263) | <0.001 |
