# Supplementary material for: Using Domain Adaptation and Inductive Transfer Learning to Improve Patient Outcome Prediction in the Intensive Care Unit: Retrospective Observational Study
Source: J Med Internet Res. 2024 Aug 21;26:e52730. doi: 10.2196/52730 (PMC11375375; doi:10.2196/52730)
Supplement: Multimedia Appendix 4 [file jmir_v26i1e52730_app4.docx]

P-values are from Wilcoxon rank sum tests. ITL: inductive transfer learning; DA: domain adaptation; FCNN: fully-connected neural network; MAE: mean absolute error; MSE: mean squared error.

| **Model** | **Data set %** | **MAE (95% CI)** | **p-value** | **MSE (95% CI)** | **p-value** |
| --- | --- | --- | --- | --- | --- |
| ITL | 1% | 13.3182 (12.6128, 13.9609) |  | 447.8828 (408.0855, 492.482) |  |
| Lasso |  | 13.7765 (13.3118, 14.2661) | <0.001 | 392.2557 (359.4263, 427.8242) | <0.001 |
| FCNN |  | 18.5363 (17.8711, 19.243) | <0.001 | 757.2068 (702.1133, 814.751) | <0.001 |
| ITL | 5% | 12.6206 (12.0632, 13.1968) |  | 430.3451 (390.3408, 473.872) |  |
| Lasso |  | 13.4951 (13.021, 13.9972) | <0.001 | 382.3238 (350.2288, 416.3966) | <0.001 |
| FCNN |  | 18.5283 (17.8824, 19.2202) | <0.001 | 757.0451 (702.3175, 813.7623) | <0.001 |
| ITL | 10% | 12.3101 (11.7013, 12.9028) |  | 421.3046 (381.0975, 464.7307) |  |
| Lasso |  | 13.4749 (12.9998, 13.9555) | <0.001 | 380.2511 (348.6, 414.0115) | <0.001 |
| FCNN |  | 18.5442 (17.8751, 19.2403) | <0.001 | 757.1631 (702.179, 814.362) | <0.001 |
| ITL | 25% | 12.1049 (11.5263, 12.6837) |  | 418.9181 (379.4325, 459.8645) |  |
| Lasso |  | 13.4623 (12.9847, 13.9355) | <0.001 | 379.583 (347.903, 412.0801) | <0.001 |
| FCNN |  | 18.5371 (17.8755, 19.236) | <0.001 | 756.5469 (700.7609, 814.4013) | <0.001 |
| ITL | 50% | 12.0137 (11.4506, 12.5691) |  | 418.237 (378.2952, 457.5163) |  |
| Lasso |  | 13.4962 (13.0424, 13.9672) | <0.001 | 379.8112 (348.5856, 412.7653) | <0.001 |
| FCNN |  | 18.5258 (17.8437, 19.2152) | <0.001 | 755.9186 (700.3882, 813.4046) | <0.001 |
| ITL | 75% | 11.9479 (11.3998, 12.5178) |  | 413.234 (373.2978, 455.0457) |  |
| Lasso |  | 13.5095 (13.0379, 13.9729) | <0.001 | 379.9352 (348.6542, 412.6511) | <0.001 |
| FCNN |  | 18.517 (17.8388, 19.2102) | <0.001 | 755.8523 (699.8628, 813.0433) | <0.001 |
| ITL | 100% | 11.9513 (11.4536, 12.4782) |  | 417.7272 (381.3438, 456.8444) |  |
| Lasso |  | 13.5014 (13.0811, 13.9509) | <0.001 | 380.6649 (349.7556, 413.8401) | <0.001 |
| FCNN |  | 18.5338 (17.9376, 19.181) | <0.001 | 755.8648 (703.5376, 810.9559) | <0.001 |
| DA | 1% | 5.838 (5.1601, 38.4836) |  | 354.9819 (58.6926, 1133085.6145) |  |
| Lasso |  | 5.175 (4.9912, 5.3943) | <0.001 | 47.4225 (43.715, 51.2107) | <0.001 |
| FCNN |  | 9.5497 (9.2446, 10.139) | <0.001 | 148.0849 (135.763, 600.7748) | <0.001 |
| DA | 5% | 5.2217 (4.834, 27.2889) |  | 136.4261 (51.6034, 1298438.6981) |  |
| Lasso |  | 5.1059 (4.9377, 10.7306) | <0.001 | 46.8123 (43.2534, 51783.3923) | <0.001 |
| FCNN |  | 9.5402 (9.2311, 10.1744) | <0.001 | 164.3962 (135.0646, 710.5129) | <0.001 |
| DA | 10% | 5.1063 (4.7518, 10.6924) |  | 96.7196 (49.7365, 50404.2387) |  |
| Lasso |  | 5.0698 (4.8954, 16.2211) | 0.0193 | 46.599 (42.822, 195165.8377) | <0.001 |
| FCNN |  | 9.417 (9.1493, 10.2042) | <0.001 | 140.7737 (132.3352, 1566.0307) | <0.001 |
| DA | 25% | 4.9109 (4.5982, 7.389) |  | 119.516 (46.2212, 7390.3824) |  |
| Lasso |  | 5.0491 (4.8903, 6.457) | <0.001 | 46.3334 (42.7066, 5211.7219) | <0.001 |
| FCNN |  | 9.2677 (9.0162, 9.6332) | <0.001 | 137.5137 (129.4385, 340.618) | <0.001 |
| DA | 50% | 4.901 (4.5394, 9.7808) |  | 191.6203 (44.8218, 35626.6159) |  |
| Lasso |  | 5.1365 (4.8967, 16.6908) | <0.001 | 51.5395 (43.3937, 182712.103) | <0.001 |
| FCNN |  | 9.2424 (8.9877, 9.536) | <0.001 | 137.1585 (128.8273, 147.6751) | <0.001 |
| DA | 75% | 5.0084 (4.4945, 9.3259) |  | 495.9654 (45.1701, 32362.5047) |  |
| Lasso |  | 5.2403 (4.9051, 15.1199) | <0.001 | 75.1788 (43.7092, 144662.7752) | <0.001 |
| FCNN |  | 9.2385 (8.9739, 9.52) | <0.001 | 136.7045 (128.7169, 150.0491) | <0.001 |
| DA | 100% | 5.5064 (4.5919, 7.8251) |  | 2087.0465 (42.2608, 8212.7642) |  |
| Lasso |  | 6.3074 (4.9256, 10.0284) | <0.001 | 5174.2352 (44.0934, 20553.6184) | <0.001 |
| FCNN |  | 9.235 (8.9955, 9.4768) | <0.001 | 136.4476 (128.9272, 144.049) | <0.001 |
