# Supplementary material for: Using Domain Adaptation and Inductive Transfer Learning to Improve Patient Outcome Prediction in the Intensive Care Unit: Retrospective Observational Study
Source: J Med Internet Res. 2024 Aug 21;26:e52730. doi: 10.2196/52730 (PMC11375375; doi:10.2196/52730)
Supplement: Multimedia Appendix 5 [file jmir_v26i1e52730_app5.docx]

P-values are from Wilcoxon rank sum tests. ITL: inductive transfer learning; LR: logistic regression; FCNN: fully-connected neural network; AUC: area under the receiver operating characteristic curve.

| Model | Data set % | Balanced Accuracy (95% CI) | p-value | AUC (95% CI) | p-value | Accuracy (95% CI) | p-value | Precision (95% CI) | p-value | Recall (95% CI) | p-value |
| --- | --- | --- | --- | --- | --- | --- | --- | --- | --- | --- | --- |
| ITL | 1% | 0.7400 (0.7158, 0.7674) |  | 0.8327 (0.8098, 0.8517) |  | 0.7965 (0.7696, 0.8182) |  | 0.4547 (0.4110, 0.5032) |  | 0.6513 (0.5927, 0.7307) |  |
| LR |  | 0.6548 (0.6334, 0.6743) | <0.001 | 0.8490 (0.8320, 0.8636) | <0.001 | 0.8545 (0.8436, 0.8662) | <0.001 | 0.6990 (0.6472, 0.7473) | <0.001 | 0.3421 (0.2983, 0.3817) | <0.001 |
| FCNN |  | 0.6834 (0.6587, 0.7189) | <0.001 | 0.7684 (0.7312, 0.7956) | <0.001 | 0.7703 (0.7299, 0.8070) | <0.001 | 0.4041 (0.3445, 0.4715) | <0.001 | 0.5543 (0.4668, 0.6203) | <0.001 |
| ITL | 5% | 0.7537 (0.7323, 0.7775) |  | 0.8388 (0.821, 0.8602) |  | 0.7810 (0.7625, 0.8057) |  | 0.4352 (0.4031, 0.4784) |  | 0.7080 (0.6546, 0.7772) |  |
| LR |  | 0.6928 (0.6663, 0.7140) | <0.001 | 0.8480 (0.8302, 0.8629) | <0.001 | 0.8553 (0.8444, 0.8670) | <0.001 | 0.6482 (0.5995, 0.6962) | <0.001 | 0.4379 (0.3827, 0.4814) | <0.001 |
| FCNN |  | 0.7235 (0.6963, 0.7514) | <0.001 | 0.8097 (0.7840, 0.8372) | <0.001 | 0.7935 (0.7673, 0.8138) | <0.001 | 0.4466 (0.3963, 0.4940) | <0.001 | 0.6143 (0.5639, 0.6822) | <0.001 |
| ITL | 10% | 0.7593 (0.7370, 0.7799) |  | 0.8453 (0.8267, 0.8608) |  | 0.779 (0.7531, 0.8032) |  | 0.4332 (0.3951, 0.4765) |  | 0.7289 (0.6662, 0.7864) |  |
| LR |  | 0.7157 (0.6947, 0.7398) | <0.001 | 0.8477 (0.8317, 0.8636) | <0.001 | 0.8464 (0.8347, 0.8591) | <0.001 | 0.5863 (0.5415, 0.6303) | <0.001 | 0.5099 (0.4673, 0.5600) | <0.001 |
| FCNN |  | 0.725 (0.6981, 0.7494) | <0.001 | 0.8180 (0.7952, 0.8400) | <0.001 | 0.7922 (0.7686, 0.8225) | <0.001 | 0.4462 (0.4015, 0.5114) | <0.001 | 0.6217 (0.5205, 0.6822) | <0.001 |
| ITL | 25% | 0.7615 (0.7429, 0.7831) |  | 0.8488 (0.8322, 0.8641) |  | 0.7782 (0.7497, 0.7965) |  | 0.4322 (0.3945, 0.4672) |  | 0.7396 (0.6968, 0.7803) |  |
| LR |  | 0.7426 (0.7226, 0.7617) | <0.001 | 0.8492 (0.8325, 0.8643) | 0.4756 | 0.8291 (0.8159, 0.8431) | <0.001 | 0.5233 (0.4850, 0.5637) | <0.001 | 0.6063 (0.5692, 0.6428) | <0.001 |
| FCNN |  | 0.7490 (0.7272, 0.7711) | <0.001 | 0.8379 (0.8188, 0.8539) | <0.001 | 0.7749 (0.7497, 0.7965) | <0.001 | 0.4255 (0.3887, 0.4660) | <0.001 | 0.7083 (0.6619, 0.7698) | <0.001 |
| ITL | 50% | 0.7639 (0.7430, 0.7855) |  | 0.8520 (0.8359, 0.8671) |  | 0.7721 (0.7197, 0.7973) |  | 0.4254 (0.3694, 0.4668) |  | 0.7556 (0.6912, 0.8177) |  |
| LR |  | 0.7484 (0.7286, 0.7680) | <0.001 | 0.8486 (0.8309, 0.8635) | <0.001 | 0.8141 (0.8009, 0.8278) | <0.001 | 0.4888 (0.4561, 0.5266) | <0.001 | 0.6454 (0.6077, 0.6838) | <0.001 |
| FCNN |  | 0.7617 (0.7425, 0.7802) | <0.001 | 0.8500 (0.8345, 0.8646) | <0.001 | 0.7749 (0.7444, 0.7953) | <0.001 | 0.4274 (0.3890, 0.4651) | <0.001 | 0.7427 (0.7007, 0.7889) | <0.001 |
| ITL | 75% | 0.7669 (0.7483, 0.7843) |  | 0.8562 (0.8387, 0.8700) |  | 0.7785 (0.7538, 0.8021) |  | 0.4338 (0.3977, 0.4762) |  | 0.7491 (0.7042, 0.7988) |  |
| LR |  | 0.7514 (0.7325, 0.7696) | <0.001 | 0.8512 (0.8353, 0.8655) | <0.001 | 0.8062 (0.7935, 0.8187) | <0.001 | 0.4734 (0.4423, 0.5082) | <0.001 | 0.6647 (0.6261, 0.7015) | <0.001 |
| FCNN |  | 0.7646 (0.7463, 0.7842) | <0.001 | 0.8543 (0.8383, 0.8690) | <0.001 | 0.7757 (0.7564, 0.7983) | <0.001 | 0.4298 (0.3988, 0.4717) | <0.001 | 0.7474 (0.7051, 0.7915) | 0.1259 |
| ITL | 100% | 0.7692 (0.7509, 0.7860) |  | 0.8565 (0.8409, 0.8711) |  | 0.7668 (0.7538, 0.7803) |  | 0.4206 (0.3941, 0.4507) |  | 0.7728 (0.7384, 0.8029) |  |
| LR |  | 0.7531 (0.7334, 0.7710) | <0.001 | 0.8511 (0.8353, 0.8664) | <0.001 | 0.8032 (0.7902, 0.8154) | <0.001 | 0.4689 (0.4388, 0.5015) | <0.001 | 0.6749 (0.6399, 0.7095) | <0.001 |
| FCNN |  | 0.7623 (0.7439, 0.7784) | <0.001 | 0.8515 (0.8363, 0.8666) | <0.001 | 0.7566 (0.7434, 0.7706) | <0.001 | 0.4081 (0.3817, 0.4372) | <0.001 | 0.7713 (0.7382, 0.8003) | <0.001 |
