# Supplementary material for: Using Domain Adaptation and Inductive Transfer Learning to Improve Patient Outcome Prediction in the Intensive Care Unit: Retrospective Observational Study
Source: J Med Internet Res. 2024 Aug 21;26:e52730. doi: 10.2196/52730 (PMC11375375; doi:10.2196/52730)
Supplement: Multimedia Appendix 6 [file jmir_v26i1e52730_app6.docx]

P-values are from Wilcoxon rank sum tests. ITL: inductive transfer learning; LR: logistic regression; FCNN: fully-connected neural network; AUC: area under the receiver operating characteristic curve.

| Model | Data set % | Balanced Accuracy (95% CI) | p-value | AUC (95% CI) | p-value | Accuracy (95% CI) | p-value | Precision (95% CI) | p-value | Recall (95% CI) | p-value |
| --- | --- | --- | --- | --- | --- | --- | --- | --- | --- | --- | --- |
| ITL | 1% | 0.6434 (0.6006, 0.6888) |  | 0.7103 (0.6606, 0.7484) |  | 0.7433 (0.6898, 0.7843) |  | 0.284 (0.237, 0.333) |  | 0.4945 (0.4055, 0.6375) |  |
| LR |  | 0.5467 (0.5154, 0.5732) | <0.001 | 0.7301 (0.694, 0.7611) | <0.001 | 0.8545 (0.8413, 0.8671) | <0.001 | 0.4773 (0.3509, 0.602) | <0.001 | 0.1153 (0.0427, 0.1708) | <0.001 |
| FCNN |  | 0.6222 (0.5757, 0.6604) | <0.001 | 0.6796 (0.6343, 0.7189) | <0.001 | 0.7668 (0.663, 0.8047) | <0.001 | 0.2823 (0.2274, 0.3517) | 0.9993 | 0.4115 (0.3143, 0.6412) | <0.001 |
| ITL | 5% | 0.6636 (0.6273, 0.6935) |  | 0.7259 (0.6779, 0.7588) |  | 0.7436 (0.7096, 0.7685) |  | 0.2918 (0.2571, 0.3282) |  | 0.5554 (0.4488, 0.633) |  |
| LR |  | 0.5941 (0.5496, 0.6283) | <0.001 | 0.7461 (0.717, 0.7737) | <0.001 | 0.8542 (0.84, 0.8677) | <0.001 | 0.4872 (0.4056, 0.5662) | <0.001 | 0.2319 (0.1271, 0.2982) | <0.001 |
| FCNN |  | 0.6222 (0.5869, 0.6514) | <0.001 | 0.6848 (0.6504, 0.7245) | <0.001 | 0.778 (0.7436, 0.8117) | <0.001 | 0.2991 (0.2554, 0.3538) | <0.001 | 0.4032 (0.3082, 0.484) | <0.001 |
| ITL | 10% | 0.6733 (0.646, 0.7043) |  | 0.7375 (0.7086, 0.7654) |  | 0.7386 (0.7058, 0.7795) |  | 0.2988 (0.261, 0.3428) |  | 0.578 (0.4881, 0.66) |  |
| LR |  | 0.6341 (0.6088, 0.6597) | <0.001 | 0.7527 (0.7253, 0.7794) | <0.001 | 0.8488 (0.8337, 0.8649) | <0.001 | 0.4674 (0.402, 0.5435) | <0.001 | 0.3333 (0.2778, 0.389) | <0.001 |
| FCNN |  | 0.6439 (0.6118, 0.6827) | <0.001 | 0.7053 (0.6738, 0.7473) | <0.001 | 0.774 (0.7345, 0.8022) | <0.001 | 0.3087 (0.2641, 0.3601) | <0.001 | 0.4615 (0.3771, 0.5614) | <0.001 |
| ITL | 25% | 0.6871 (0.66, 0.7147) |  | 0.7511 (0.7212, 0.7812) |  | 0.7436 (0.7178, 0.7729) |  | 0.3043 (0.2689, 0.3445) |  | 0.6075 (0.5395, 0.6774) |  |
| LR |  | 0.6666 (0.6405, 0.6918) | <0.001 | 0.7595 (0.7323, 0.7842) | <0.001 | 0.8271 (0.8088, 0.845) | <0.001 | 0.4088 (0.3546, 0.4667) | <0.001 | 0.4398 (0.3865, 0.4978) | <0.001 |
| FCNN |  | 0.6686 (0.635, 0.6993) | <0.001 | 0.7289 (0.696, 0.7572) | <0.001 | 0.7556 (0.7392, 0.7732) | <0.001 | 0.3053 (0.2732, 0.3418) | 0.0827 | 0.5478 (0.4707, 0.6143) | <0.001 |
| ITL | 50% | 0.6993 (0.6705, 0.7231) |  | 0.7643 (0.7401, 0.7895) |  | 0.7446 (0.7225, 0.7802) |  | 0.3127 (0.2773, 0.3574) |  | 0.6307 (0.566, 0.6883) |  |
| LR |  | 0.6872 (0.6621, 0.7138) | <0.001 | 0.764 (0.7381, 0.7873) | 0.3418 | 0.8079 (0.7918, 0.8239) | <0.001 | 0.3792 (0.338, 0.4217) | <0.001 | 0.5175 (0.4647, 0.5762) | <0.001 |
| FCNN |  | 0.6936 (0.6693, 0.7197) | <0.001 | 0.759 (0.7348, 0.7854) | <0.001 | 0.7485 (0.697, 0.7726) | <0.001 | 0.3105 (0.2702, 0.3498) | <0.001 | 0.6208 (0.5536, 0.6957) | <0.001 |
| ITL | 75% | 0.7053 (0.6803, 0.73) |  | 0.7718 (0.746, 0.7957) |  | 0.7569 (0.7263, 0.7789) |  | 0.3237 (0.2849, 0.3643) |  | 0.6335 (0.5837, 0.6891) |  |
| LR |  | 0.6972 (0.6702, 0.7224) | <0.001 | 0.7665 (0.7411, 0.7908) | <0.001 | 0.7994 (0.7842, 0.8132) | <0.001 | 0.3695 (0.3315, 0.4082) | <0.001 | 0.5538 (0.5021, 0.6009) | <0.001 |
| FCNN |  | 0.7013 (0.6763, 0.7272) | <0.001 | 0.7699 (0.7451, 0.7962) | <0.001 | 0.7474 (0.7061, 0.7805) | <0.001 | 0.3152 (0.2773, 0.3546) | <0.001 | 0.6346 (0.5605, 0.7123) | 0.3422 |
| ITL | 100% | 0.6933 (0.6701, 0.717) |  | 0.7762 (0.7531, 0.7999) |  | 0.7754 (0.7625, 0.7896) |  | 0.3373 (0.3047, 0.3724) |  | 0.5772 (0.5315, 0.625) |  |
| LR |  | 0.7007 (0.6768, 0.7257) | <0.001 | 0.7652 (0.74, 0.7893) | <0.001 | 0.7858 (0.7723, 0.7994) | <0.001 | 0.3531 (0.3199, 0.3884) | <0.001 | 0.5816 (0.5343, 0.6253) | <0.001 |
| FCNN |  | 0.6968 (0.6738, 0.7212) | <0.001 | 0.7694 (0.7448, 0.7938) | <0.001 | 0.7663 (0.7528, 0.7808) | <0.001 | 0.33 (0.2977, 0.3628) | <0.001 | 0.5983 (0.5535, 0.6438) | <0.001 |
